# From competition to complementarity: narrative review of laser interstitial thermal therapy, focused ultrasound, and radiofrequency ablation in minimally invasive neurosurgery

**DOI:** 10.1097/MS9.0000000000004916

**Published:** 2026-04-07

**Authors:** Tirath Patel, Hamza Yousuf Ibrahim, Tanzeela Khan, Tehreem Tabrez, Sara Hussain, Alishba Fatima, Aaliya Junaid Siddiqui, Nikhilesh Anand

**Affiliations:** a Department of Surgery, Trinity Medical Sciences University School of Medicine, Kingstown, Saint Vincent and the Grenadines; b Department of Surgery , Jinnah Medical and Dental College, Karachi, Pakistan; c Department of Surgery, Dow Medical College, Karachi, Pakistan; d Department of Medical Education, University of Texas Rio Grande Valley, Edinburg, Texas, USA

**Keywords:** epilepsy, focused ultrasound (FUS), glioma, laser interstitial thermal therapy (LITT), movement disorders, radiofrequency ablation (RFA)

## Abstract

Minimally invasive neurosurgical techniques have emerged as a transformative paradigm, offering alternatives to conventional open approaches. The demand for safer, less invasive procedures accelerated the development of laser interstitial thermal therapy (LITT), focused ultrasound (FUS), and radiofrequency ablation (RFA), each employing a distinct mechanism with unique clinical implications. The objective was to critically evaluate and contextualize LITT, FUS, and RFA with emphasis on their comparative efficacy, safety, and roles as competing or complementary technologies in minimally invasive neurosurgery. Literature published between 2005 and 2025 was identified through PubMed, Scopus, and Web of Science, using keywords “LITT,” “FUS,” “RFA,” “minimally invasive neurosurgery,” “epilepsy,” “glioma,” and “movement disorders.” Eligible English studies included clinical trials, systematic reviews, meta-analyses, and large observational studies. The extracted data were synthesized narratively, focusing on clinical indications, efficacy, safety, and patient-centered outcomes. Evidence highlights the roles of LITT, FUS, and RFA across tumors, epilepsy, and movement disorders with differing efficacy and safety indications. LITT is most effective in gliomas and metastases, FUS shows its strongest evidence in movement disorders, whereas LITT and RFA via stereo-electroencephalography-guided thermocoagulation remain relevant in epilepsy. Reported safety outcomes include edema with LITT, skull heating with FUS, and hemorrhage with RFA. Patient-centered outcomes across modalities suggest shorter recovery periods, improved cosmesis and quality of life, and favorable cost-effectiveness. LITT, FUS, and RFA represent complementary rather than competitive modalities in minimally invasive neurosurgery. Advances in imaging, navigation, thermal technologies, and patient-centered approaches are likely to accelerate their integration into cohesive, multimodal neurosurgical strategies.

## Introduction

Minimally invasive neurosurgery has gained considerable attention in recent years for its potential to treat various neurological disorders while preventing collateral damage. Traditional approaches like craniotomies and lobotomies require unnecessary exposure of the brain tissue, which can increase the risk of complications like infections and bleeding. In contrast, minimally invasive approaches minimize tissue damage, reduce blood loss, and shorten hospital stays[1]. Most importantly, they allow for serial ablation in a segmented manner, whereas conventional open surgeries are typically limited to a single intervention[2]. Consequently, these approaches have successfully mitigated complications and risks associated with open surgeries while providing comparable therapeutic outcomes.

Within this evolving landscape, ablative energy-based technologies have emerged as novel therapies. These include laser interstitial thermal therapy (LITT), focused ultrasound (FUS), and radiofrequency ablation (RFA). Such therapies employ therapeutic lesions to selectively destroy a targeted volume of pathological brain tissue, like brain tumors or dysfunctional brain circuits. They are progressively acknowledged in recent years due to their precision, real-time monitoring of tissue changes, and less invasive method of creating therapeutic lesions[1], making them valuable in clinical domains like epilepsy, brain tumors, functional neurosurgery, and movement disorders. Given their rising role in neurosurgery, this review aims to critically compare and contextualize these three modalities as either competing or complementary ablative technologies.

This manuscript is made compliant with the TITAN checklist to ensure transparency in the reporting of Artificial Intelligence[3].

## Methodology

We conducted this narrative review to compare three emerging minimal-invasive neurosurgery techniques: LITT, FUS, and RFA. Our focus remained on whether these emerging modalities were mutually exclusive in neurosurgery or could potentially emerge as complementary techniques in the future, particularly based on patient outcomes and other relevant criteria.

### Literature search strategy

A comprehensive literature search was performed using PubMed, Scopus, and Web of Science. Articles published in the last 20 years, from 2005 to 2025, were included in the search. Keywords used to search in the aforementioned databases included “Laser Interstitial Thermal Therapy,” “Focused Ultrasound,” “Radiofrequency Ablation,” “minimally invasive neurosurgery,” “epilepsy,” “glioma,” and “movement disorders.”

### Inclusion and exclusion criteria

Clinical trials, systematic reviews, meta-analyses, and large observational studies that were written in English were included. Studies especially focused on reporting outcomes in neurosurgical outcomes were prioritized.

Non-English studies, purely animal or *in vitro* studies (unless considered highly translational), and case reports with less than five reported patients (unless historically significant) were excluded from the search.

### Data extraction and synthesis

Data extraction was performed by two reviewers focusing on patient population, indication, patient details, outcomes, and follow-up. Citation management software was used to organize references. The data were analyzed with the aim of conceptual integration with a focus on comparative discussion on the techniques. Since this is a narrative and not a systematic review, it did not provide any formal risk-of-bias evaluation or quantitative meta-analysis.

Supplemental Digital Content Table S1, available at: http://links.lww.com/MS9/B112, provides an overview of the databases that were searched, the timeframe of the search, the key terms employed, the criteria for inclusion and exclusion, and the synthesis method used for this narrative review.

## Historical and technological foundations

### Laser interstitial thermal therapy (LITT)

Laser interstitial thermotherapy, also called stereotactic laser ablation, is a minimally invasive therapeutic approach that utilizes fiber optic catheter-based lasers to deliver focused thermal energy, thereby achieving local tissue destruction[4]. One of the earliest attempts at laser thermal delivery in neurosurgery was reported by Bown *et al* in 1983. They utilized a neodymium-doped yttrium aluminum (ND: YAG) laser for an experimental tumor model, laying the groundwork for subsequent refinements in laser-based neurosurgical interventions[4]. Widespread adoption of this technology was hindered by several setbacks, such as a lack of feedback control and precise thermal monitoring, and therefore, the extent of ablation[5].

**HIGHLIGHTS**

- LITT, FUS, and RFA provide safer alternatives to open neurosurgery, reducing morbidity, shortening recovery times, and enhancing patient-centered outcomes.
- FUS is most effective for movement disorders; LITT has strong evidence in gliomas and metastases, while both LITT and RFA remain valuable for epilepsy.
- Each modality carries distinct risks: hemorrhage with RFA, skull heating with FUS, and cerebral edema with LITT, requiring careful patient selection.
- LITT, FUS, and RFA improve quality of life by minimizing hospital stays, ensuring superior cosmetic outcomes, and offering favorable cost-effectiveness compared with open surgery.
- LITT, FUS, and RFA function as complementary tools within a multimodal, personalized neurosurgical approach, supported by advances in imaging, navigation, and artificial intelligence.

These limitations prevented its early neurosurgical use. However, the procedure later evolved with the integration of two technological developments. First, the introduction of efficient catheter-based lasers, which were capable of delivering near-infrared radiation, allowed for predictable ablation of targeted tissue. The second revolutionary change was with the emergence of efficient temperature monitoring technology, MRI thermometry. It facilitated real-time thermal monitoring of the procedure and direct visual feedback regarding the induced thermal tissue damage[5]. This enabled precise delineation of the target volume for thermal energy delivery while also incorporating safety cutoff markers to avoid damage to functionally important brain structures[5]. These advancements optimized the safety associated with stereotactic ablation, hence epileptogenic foci and epidural spinal metastases^[^4,5^]^. For instance, a recent meta-analysis on the safety and efficacy of LITT illustrated the highest rate of seizure freedom (i.e., 55%) and the lowest rate of complication (i.e., 2.3%) in single-arm studies of mesial temporal lobe epilepsy (MTLE)[6].

### Mechanism

A laser is a source of non-ionizing radiation that delivers coherent, monochromatic, and collimated light energy in the form of a focused beam[4]. The ability of lasers to achieve stereotactic ablation derives from two principal phenomena.

1. Absorption: As soon as laser photons collide with the molecules in target tissue, the photon energy gets absorbed and converted to heat energy. The energy transfer and conversion result in photothermal heating and subsequent thermal ablation of brain tissue.

2. Scatter: Deviation in the photons’ trajectory after interacting with different particles in tissue results in a uniform spatial distribution of light and heat energy[7]. MRI-guided LITT transmits laser light interstitially through flexible fiber-optic wires, coupling the generator to patient tissue[7] and achieves thermal coagulation and ablation by rapidly heating the tissue beyond a threshold level, consequently damaging cellular DNA and resulting in gliosis and necrosis[4].

However, it is crucial to deliver thermal energy in a predictable pattern without causing overheating. MRI thermometry has successfully allowed for temperature feedback control and management. Moreover, with the innovation of the catheter cooling system, we have been able to overcome the thermal buildup, thereby allowing better treatment outcomes[4].

### Focused ultrasound (FUS)

Ultrasound is a widely used diagnostic and monitoring modality in the medical field that utilizes acoustic energy for real-time visualization of soft tissues inside the body. However, in recent years, investigations have been conducted on its therapeutic use. According to preliminary data, high-intensity acoustic energy, when focused on specific areas, can produce well-defined lesions in deep tissues, including the brain, without damaging surrounding structures. The idea was first realized in 1935, when Gruetzmacher designed a curved quartz plate that could concentrate ultrasound beams at a specific point[8]. It was also reported that four FUS beams were used for treating various human patients with movement disorders, particularly Parkinson’s disease[9]. Although the clinical applications appeared successful, its relevance as a minimally invasive surgery was limited, as it required craniotomy to deliver ultrasound energy[9].

With the advent of multiple phased-array ultrasound transducers, algorithms accounting for skull penetration by sound waves, and magnetic resonance (MR) thermometry for target monitoring of the thermal ablation field, brain lesioning with sub-millimeter precision became possible[10]. Thus, FUS has established itself as a promising noninvasive ablative technique.

Emerging clinical applications of FUS include its successful implementation in the treatment of Parkinson’s disease, essential tremor, obsessive-compulsive disorder, and chronic neuropathic pain[11]. For instance, a multicenter trial in 2016 demonstrated improvement in hand tremor scores from 18.1 points at baseline to 9.6 at 3 months in the MR-guided FUS (MRgFUS) thalamotomy group compared with the sham group. Moreover, the therapeutic benefit persisted for 12 months with tolerable side effects. This technology has expanded its applicability in broader neurosurgical fields, with ongoing trials showcasing its potential in the management of epilepsy, brain tumors, and blood–brain barrier modulation for enhanced drug delivery. For example, a recent meta-analysis of 24 clinical and preclinical studies showed FUS as a safe and feasible approach for the management of epileptic seizures, with a 13% adverse event rate[12].

### Mechanism

Ultrasound is a type of mechanical wave that propagates at a frequency beyond the human threshold for hearing, i.e., 20–20 000 Hz[7]. These ultrasonic waves travel through tissues, get absorbed, and produce both thermal and non-thermal effects. Thermal effects are achieved by focusing sound waves at a small target volume, the focal zone. It significantly increases the intensity, thereby causing localized energy deposition in target tissue known as sonication. The highly dense acoustic waves elevate the temperature of the tissues up to 60°C, inducing cytotoxic effects and resultant necrotic lesions measuring 50–300 mm in volume. Appropriate intersonication gaps are critical to avoid tissue boiling and bubble formation, which can distort the ultrasound field. Non-thermal or mechanical effects of ultrasound should be considered while discussing FUS, as its effectiveness in achieving ablation mainly stems from its ability to induce mechanical effects.

FUS can induce oscillation of trapped gas particles inside our tissues, in a process called cavitation. Mechanical pressure causes bubbles to undergo compression and rarefaction at high intensities; these bubbles collapse violently, generating heat, shear stress, and micro-jets of liquid. This can damage cell membranes, trigger apoptosis, and cause DNA degradation by endonucleases[13]. Validity disorders increase minimally, reduce, allow, and cause one-time complications, and are real-time, making this review aim at them. Catheter-based applications, such as limitations, near-infrared, and propagation efficiency, have been improved through innovations like MRI and MRI thermometry for precise target localization, efficient treatment planning, and simultaneous real-time monitoring of thermal changes[13].

### Radiofrequency ablation (RFA)

RFA is one of the earliest ablative therapeutic techniques that utilizes high-frequency alternating current (AC) to induce protein denaturation and subsequent cell death within targeted tissues[14]. In the early 1950s, Spiegel and Wycis pioneered stereotactic neurosurgery, where they initially used direct current (DC) for thermal ablation to treat epilepsy and movement disorders. Later, due to uncertainty in lesion size and uncontrolled tissue destruction with DC electrolysis, the idea was largely abandoned[15]. Around the same time period, William Sweet introduced radiofrequency (RF) lesions utilizing AC to create lesions, which proved to be more precise and controllable compared to the earlier practice of DC lesions[16]. This marked the beginning of RF lesions in functional neurosurgery. The transition replaced early crude lesion techniques with precise controlled ablation, which enabled the development of reliable procedures such as pallidotomy and thalamotomy for movement disorders and amygdalotomy for the treatment of epilepsy[17]. In the following years, with technological advancements in RF lesion, stereotactic techniques, CT, and MRI, coupled with the progressive knowledge of psychiatric and movement disorders, this technology was widely accepted and became the preferred method in functional neurosurgery. According to research conducted on 14 June 2021, analyzing radiofrequency lesioning (RFL) in contemporary functional neurosurgery. RFL demonstrated improvement in mean tremor scores and UPDRS (Unified Parkinson’s Disease Rating Scale) of Parkinson’s disease patients from 4.3 to 1.1, i.e., 74.42%, and 22.6 to 11.4, i.e., 49.56%, respectively[16].

### Mechanism

RF is a type of thermal ablative procedure that causes coagulative necrosis of tumors by elevating the temperature up to 60 degrees. The underlying principle involves integration of RF electrodes to the tumor site and delivery of a high-frequency AC (400–500 kHz), which creates oscillation and agitation among ions, generating frictional heat. This phenomenon is called the Joule effect. The high temperature eventually heats the cell to death through protein denaturation and melted lipid bilayers. As a result, tumor cells degenerate, and coagulative necrosis ensues[14].

Table 1 provides the technical characteristics of LITT, FUS, and RFA.

**Table 1:** Technical characteristics of LITT, FUS, and RFA.

Features	LITT	FUS	RFA
Energy source	Laser-based thermal energy[4]	High-intensity FUS waves[8]	High-frequency AC[16]
Mechanism of action	Thermal coagulation and ablation via catheter-based laser[4]	Highly dense acoustic wave focused to produce cytotoxic effects[13]	AC creates oscillation and agitation among ions, generating frictional heat leading to coagulative necrosis[16]
Guidance and imaging	MRI-guided and MRI thermometry for temperature feedback control[4]	MRI-guided for precise target localization, MR thermometry for real-time thermal monitoring[13]	CT-, MRI-, or ultrasound-guided^[^15,17^]^
Invasiveness	Minimally invasive; catheter-based insertion of laser source[5]	Noninvasive, no incision required[8]	Minimally invasive; needle electrode inserted into target tissue[17]
Delivery method	Stereo tactically placed fiber optic catheter[5]	Transcranial[8]	CT scan-based stereotactic targeting[14]
Lesion control	High precision	High precision	Lesion size depends on electrode geometry[18]
Limitation	Lesion size is limited as heat transfer is distance dependent[5]	Skull density acts as the main barrier to energy transfer[8]	Limited by heat diffusion due to the heat sink effect[16]
Clinical application	Epilepsy, glioblastomas, spinal tumors[5]	Essential tremor, Parkinson’s disease, neuropathic pain, obsessive compulsive disorder[8]	Movement disorder, epilepsy, various types of cancer, and cardiovascular disease mechanisms[16]

## Clinical applications

Minimally invasive ablative procedures have become a major focus in neurosurgery due to their expanding clinical applications. While traditional open neurosurgical techniques remain widely practiced, they are associated with considerable morbidity and mortality, especially in patients with deep-seated or refractory medical disease[19]. Over time, the ablative technologies gradually transitioned from experimental tools to clinically adopted therapies. This progression is reflected in the chronological development of three major modalities: RFA, FUS, and LITT.

RFA was first introduced in 1953, establishing its role in functional neurosurgery[16]. Though not disease-specific, RFA has continued to evolve, leading to an FDA-approved system in 2023[20]. FUS was then followed in 1959 with Meyers and Fry’s work on movement disorders[21]. Subsequent refinements in FUS led to FDA approvals for essential tremor in 2016[22] and tremor-predominant Parkinson’s disease in 2018[23]. In parallel, LITT emerged in 1990 for brain tumors in Japan, with the first FDA-approved MR-guided LITT procedure for epileptogenic foci reported in 2012[24]. Collectively, these milestones show the evolution of ablative technologies, with current research primarily centered around three clinical domains: neurooncology, epilepsy, and functional neurosurgery.

### Gliomas and brain metastases

High-grade gliomas often recur, even after multimodal treatment like chemotherapy or open surgical procedure. Many patients become ineligible for repeat craniotomy due to inaccessible deep-seated tumor location, proximity to eloquent cortex, significant comorbidities, poor functional status, or reluctance to undergo another open procedure[25]. In this context, LITT has emerged as a minimally invasive cytoreductive option. Studies have demonstrated LITT’s efficacy in controlling recurrent gliomas and brain metastases. For instance, a phase-I trial compared the effect of Avelumab alone and Avelumab as an adjunct therapy along with LITT in recurrent glioblastoma, reporting a prolonged progression-free survival (PFS) in the combination group[26]. Furthermore, repeat LITT has also provided durable disease control in gliomas and brain metastases, with minimal pre- and post-operative complications[27]. Beyond survival metrics, functional preservation remains a critical consideration in recurrent gliomas. A single-center cohort reported a nearly 60% functional preservation rate, as indicated by Karnofsky Performance Status post-procedure, while 41% patients with ill-defined lesions experienced functional decline. This highlights a contrast between the achievement of maximal ablation and the preservation of neurological function, suggesting that well-defined lesions are optimal candidates[25].

In addition to LITT, FUS offers an alternative mechanism by enabling blood–brain barrier disruption and enhancing drug penetration with minimal adverse effects^[^28,29^]^. Its role in thermal ablation is still under investigation with early preclinical studies, suggesting the potential of high-intensity FUS in thermal ablation[30]. Similarly, RFA has also demonstrated effectiveness in deep-seated brain neoplasms, with survival rates of 80% in metastases and 60% in gliomas[30]. However, clinical studies on RFA in neurooncology remain limited, highlighting the need for further investigations.

### Epilepsy

Drug-resistant epilepsy remains a challenge, despite the appropriate pharmacological intervention, which becomes ineffective for patients, ultimately leading them towards surgical options[31]. Among the minimally invasive techniques, LITT has emerged as a leading minimally invasive option with multicenter studies showing ENGEL I seizure-free rates of 58% in MTLE[32] and a systematic review showing a seizure freedom rate of 65.9% in hypothalamic hamartomas (HHs)[33]. Likewise, FUS also demonstrated a significant reduction in seizure frequency in drug-resistant epilepsy patients^[^34,35^]^, yet its studies remain limited due to small sample size. By contrast, RFA possesses a greater amount of evidence, particularly stereo-electroencephalography (SEEG)-guided procedures, reporting seizure freedom rates ranging between 24 and 40^[^36,37^]^. Notably, in HH patients, RFA has achieved an even higher seizure freedom rate of 70.4%[38].

### Movement disorders (Parkinson’s and essential tremors)

Movement disorders, including Parkinson’s and essential tremors, significantly reduce quality of life (QoL), sometimes even after suitable pharmacological regimens. Minimally invasive ablative approaches are therefore being increasingly explored to maintain the QoL among these patients. Among these, FUS has shown the strongest evidence. Randomized trials report a significant improvement in movement disorder society-sponsored revision of the UPDRS and Unified Dyskinesia rating scale scores, reflecting motor skills improvement in Parkinson’s^[^39,40^]^, and FDA-approved thalamotomy for essential tremors reporting a tremor reduction of more than 60%[41]. In contrast, RFA provides a promising effective rate of about 90%, although it shows a higher prevalence of adverse events^[^42,43^]^. Meanwhile, LITT, supported by a pilot study, shows a substantial improvement in tremors and remains a novel therapy in this regard; hence, it needs to be investigated further[44].

**Figure 1.**
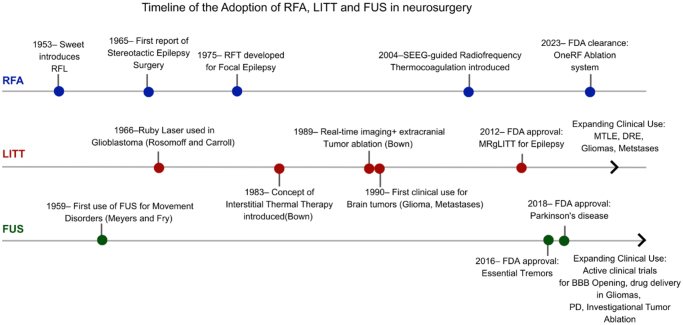
Timeline of the adoption of RFA, LITT, and FUS in neurosurgery. This schematic timeline illustrates the introduction and early use of RFA, LITT, and FUS, their FDA approvals, and the expansion of indications.

Table 2 shows clinical indications and evidence strength. Figure 1 shows a timeline of the adoption of RFA, LITT, and FUS in Neurosurgery. This schematic timeline illustrates the introduction and early use of RFA, LITT, and FUS, their FDA approvals, and the expansion of indications.

**Table 2:** Clinical indications and evidence strength.

Indications	Modality	Evidence
Recurrent gliomas/metastases	LITT	Moderate to high (RCTs[26], cohorts[25], and case series[27])
	FUS	Low (early studies, BBB disruption focus^[^28,29^]^)
	RFA	Low (limited case studies[30])
Epilepsy	LITT	Moderate (multicenter studies[32] for MTLE) to high (SRMA and retrospective studies[33] for HH)
	FUS	Low (pilot studies[35] and cohorts[34] with small sample size)
	RFA	Moderate (SEEG-guided retrospective studies^[^36,38^]^ and systematic reviews[37])
Functional neurosurgery (Parkinson’s and essential tremors)	LITT	Low (early evidence[44])
	FUS	High (FDA-approved RCTs^[^39,41^]^)
	RFA	Moderate (systematic reviews and cohorts^[^42,43^]^; reports more adverse events)

LITT, laser interstitial thermal therapy; FUS, focused ultrasound; RFA, radiofrequency ablation; BBB, blood brain barrier; SRMA, systemataic review and meta analysis.

## Comparative efficacy and safety

### Short-term versus long-term efficacy

Researchers have explored the effectiveness of newly emerging minimally invasive techniques like LITT, RFA, and FUS for different etiologies. Evidence from these studies suggests varying patterns of short-term outcomes and long-term durability across the modalities, which are discussed below.

### Laser interstitial thermal therapy (LITT)

Patients suffering from drug-resistant epilepsy and glioma have shown favorable short- and long-term outcomes when treated with the LITT technique. For instance, recurrent glioblastoma patients showed tumor reduction on radiograph with higher progression-free survival and overall survival (OS) when treated with LITT[45].

It was observed that when high-grade glioma patients were treated with only LITT, they showed a median PFS of 5.1 months and an OS of 68%, while regrowth of the tumor occurring in 71% of patients in a short time was noticed[46]. However, when post-treatment tumor resection was done in similar patients, better outcomes with an average PFS of 9.3 months and OS of 16.1 months were observed[47]. This comparison shows that the overall impact of the LITT technique was favorable, while outcomes of LITT were comparatively better when paired with post-LITT tumor resection.

For epileptic patients, Du *et al* (2017) observed that six out of seven patients reached seizure freedom in a mean follow-up of 17.7 months[48]. In addition to that, Barot *et al* showed a collective seizure-free rate of 55.2% while also highlighting that those patients with HHs showed the best results[49]. Infante *et al* (2025) also reported 85.7% of patients being seizure-free in HH[50]. These findings indicate that patients with HH are more likely to have better outcomes than those with other etiologies.

LITT treatment has given promising short-term outcomes with slight variations depending on the type of etiology and has provided durable long-term effects in both epilepsy and glioma patients.

### Radiofrequency ablation (RFA)

SEEG-guided RFTC has been successfully used for the treatment of patients with drug-resistant epilepsy. A significant reduction in seizure rate was noted shortly after the treatment. For instance, Bourdillon *et al* reported that 25% patients achieved seizure freedom while 67% responded to the therapy at 2-month follow-up[51]. Similarly, Kerezoudis *et al* mentioned that 68% patients suffering from drug-resistant epilepsy were seizure-free at 1-year follow-up, while another recent research reported an even higher seizure freedom rate of 72.4%^[^38,52^]^. These findings support that RFA can be a possible treatment option for immediate favorable outcomes in patients with drug-resistant epilepsy.

However, a gradual decrease in the effectiveness of RFA was noticed after the initial positive outcomes. For example, Bourdillon *et al* observed that the seizure control rate declined from 25% to only 7% in 10 years[51]. Correspondingly, Li *et al* also discovered that while the early promising results of seizure freedom were achieved in 72.4% patients, the rate dropped to 42.9% in 5-year extended follow-up. Also, a research finding implies that 28.1% patients showed sustained improvement over prolonged follow-ups. This summarizes that the efficacy of RFA remains limited and tends to reduce over time[53].

Overall, RFA provided remarkable short-term results, and while the long-term efficacy initially declined, RFTC was able to maintain the seizure freedom rates at optimum levels over extended follow-ups.

### Focused ultrasound (FUS)

MRgFUS thalamotomy is a minimally invasive and very effective treatment for medication-refractory tremor with proven efficacy evident in various clinical studies. For instance, A recent research by Krishna *et al* (2023) indicated that 69% of patients demonstrated improved motor movements in the FUS group, while only 32% achieved the same outcome in the sham group[41]. The results are consistent with findings of Martínez-Fernández *et al* (2023), who also reported a 52.3% decrease in motor dysfunction in 3 months after the treatment[54]. Similarly, another study reported significant improvement in hand tremor when treated with FUS, with the sham group showing very little change[55]. This shows that FUS has been successful in delivering favorable immediate results in drug-resistant tremors.

Although the evidence for the long-term durability of FUS is still evolving, the available data demonstrate impressive results. For example, a research study highlighted that the majority of patients maintained their results till 12 months post-treatment with FUS[54]. Moreover, Martínez-Fernández *et al* (2023) emphasized that most patients had sustained improved motor functions at the 36-month follow-up[55]. Reinforcing these findings, Yamamoto *et al* and Elias *et al* also demonstrated significant improvement in tremor intensity at 12 months post-treatment[55]. Summarizing the studies, MRgFUS thalamotomy offers exceptional short-term improvement for medication-refractory tremor with promising long-term durability.

### Adverse effects

While these technologies provide remarkable and promising results, some adverse results were also reported during the follow-ups. For the patients who were treated with LITT, 25% of patients were found to develop cerebral edema following the treatment[56]. Barot *et al* reported that the most common complication with the treatment of LITT was visual field deficits[50].

It was found that the RFA procedure carried a potential risk of hemorrhage, and also the patients experienced serious and often permanent neurological deficits after undergoing RFA surgery[53].

Patients who underwent FUS were reported to experience gait disturbance and sensory symptoms, which persisted for as long as 12 months in some patients. Skull heating was a technical challenge presented, which requires skull cooling during the procedure[55].

### Learning curve/reproducibility

The primary challenge with LITT was centered on having the proficiency for surgical and trajectory planning, placement of the catheter, and interpretation of real MRI during the procedure to protect against damage. In RFA, the surgeons demonstrated improved precision during the 30 cases, which points towards a definite learning curve. It was also mentioned that the technique could be considered safe to implement without compromising the quality of care[57]. During the early use of FUS, the surgeons carefully opted for patients ideal for treatment. As their expertise increased, they advanced the treatment to more complex cases. With experience, the surgeons were able to complete the procedure with more proficiency and shorter duration[58].

For FUS initially, they only treated ideal patients, then, with enough experience, treated more complex cases. As the experience increased, they completed the procedure in a shorter time with more efficiency[58].

**Figure 2.**
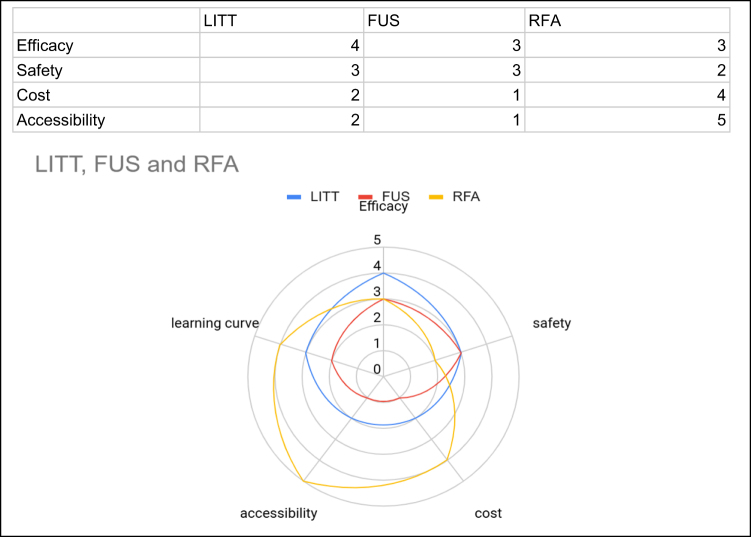
Comparison of LITT, FUS, and RFA across multiple parameters (efficacy, safety, cost, accessibility, and learning curve) using a radar chart. LITT= laser interstitial thermal therapy, FUS= Focused ultrasound, RFA = Radio-frequency ablation.

Table 3 shows comparative efficacy and safety profiles. Figure 2 shows a comparison of LITT, FUS, and RFA across multiple parameters (efficacy, safety, cost, accessibility, and learning curve) using a radar chart.

**Table 3:** Comparative efficacy and safety profiles.

	LITT	RFA	FUS
Short-term efficacy	High-grade glioma patients showed an average PFS of 9.3 months[47]	At 2-month follow-up, 25% patients were seizure-free and 67% were responders[51]	Patients undergoing FUS treatment experienced a significant reduction in hand tremor, with scores improving from 18.1 to 9.6 points on the Clinical Tremor Rating Scale[55]
	Regrowth of the tumor occurred in 71% patients[46]	62% achieved seizure freedom at 12-month follow-up[38]	
Long-term efficacy	Collective seizure-free rate of 55.2%	Only 7% stayed seizure-free and 48% stayed responders at 1 year, and just 13% continued as responders at 10 years[51]	The improvements in tremor severity and disability that were seen at 3 months follow-up were maintained at 12 months after treatment[55]
	85.7% seizure-free in HH[50]		
	New GBM cases show an 8-month median OS after LITT[45]	28.1% of patients showed sustained improvement[53]	
Safety profile	Post-treatment adverse effect rate was 19%[50]	Risk of hemorrhage and serious, and often permanent, neurological deficits[53]	Gait disturbances, neurological motor deficits, and skull heating[55]
	25% of patients developed cerebral edema		

LITT, laser interstitial thermal therapy; FUS, focused ultrasound; RFA, radiofrequency ablation.

## Patient-centered outcomes

Apart from the technical success of controlling lesions, the three procedures are evaluated based on the impact they have on the daily life of patients’ post-treatment. The QoL after receiving the treatment, the time necessary for recovery, the cosmetic appearance, and whether the treatment is outpatient or not are variables used to evaluate the effectiveness of a procedure based on a patient’s experience. Economic considerations also play a role in determining the value of these therapies. Table 4 summarizes patient-centered outcomes of LITT, FUS, and RFA.

**Table 4:** Patient-centered outcomes across LITT, FUS, and RFA.

Outcomes	LITT	FUS	RFA
QoL	Median QOLIE-31 score improved by +14.1 from baseline (51.7)[59]	The QUEST score lowered by 46% at the 3-month follow-up[60]	Immediate pain alleviation by excision of pain pathways[61]
Recovery	Brief hospitalization ~1 day[62]	Same-day discharge. Regain normal function ~7 days[63]	Fast recovery. Short hospitalization ~3–5 days[64]
Cosmesis	Small burr-hole, 5 mm or less[62]	No incision[55]	Tiny, hardly perceptible entry points[61]
Outpatient vs Inpatient	Short inpatient stays[68]	Outpatient procedure requiring 2–3 h[63]	Performed as an inpatient lasting from 15 min to 2 h[65]
Economics	30–40% cheaper than craniotomy[68]	Economically favorable for tremor treatment[66]	Generally considered a cost-effective option[61]

### Quality of life (QoL)

LITT has been linked to appreciable improvement in QoL, especially in epilepsy patients. Landazuri and colleagues reported that the median QOLIE-31 score increased by 14.1 points on follow-up from the previously measured 51.7, highlighting the worthiness of the procedure in seizure management[59]. FUS, most commonly utilized in essential tremor patients, is a procedure that shows a similar increase in QoL. In a study by Jung *et al*, patients experienced a 46% reduction in QUEST scores, reflecting an upward trend in daily functioning[60]. Although limited data are available for RFA in minimally invasive neurosurgery, it has also been identified as relieving pain immediately in a study by Eskandar *et al* and thus increasing QoL[61].

### Recovery

The effectiveness of a procedure is directly related to the time duration for recovery. In contrast to longer hospital stays typical of open neurosurgery, patients undergoing LITT need only about 1 day before discharge[62]. The hospitalization time frame for FUS is shorter than LITT, with patients being discharged the same day and returning to normal daily activities within a week[63]. RFA has a longer recovery period when compared with LITT and FUS, with an average of 3–5 days of hospitalization, but being minimally invasive, it is also considered a speedy recovery[64].

### Cosmesis

Cosmetic outcome is an important factor when comparing the favorability of procedures for patients. As minimally invasive approaches, all three techniques have shown excellent results in this regard, leaving little to no visible scarring. For example, LITT requires only a small burr-hole incision of 5 mm or less[62], while FUS achieves its effect without any incision, making it the most favorable from a cosmetic standpoint[55]. Likewise, scars from RFA are barely noticeable, leaving only tiny entrance sites behind[61].

### Outpatient versus inpatient

There is a difference in the hospital setting among these three procedures. In comparison with traditional craniotomy, LITT necessitates a shorter hospital stay but is still an inpatient procedure[65]. FUS, on the other hand, is a completely outpatient treatment that takes at most 2–3 h[63]. In terms of convenience, RFA is placed in the middle of the two, as the procedure itself needs a short duration, usually 15 min to 2 h, but requires inpatient monitoring[66].

### Economics

Cost-effectiveness is a crucial factor influencing a patient’s choice of treatment. Despite some variability in data comparing LITT, FUS, and RFA with traditional surgical approaches, the general trend suggests that minimally invasive techniques are economically favorable. In support of this cost-effectiveness, Tyagi *et al* reported in their study that LITT is safer and 30–40% cheaper than craniotomy[65]. Likewise, FUS has also been evaluated and proved to be cost-effective in research by Ravikumar *et al*[67]. Despite being less researched in this area, RFA is generally regarded as affordable when compared with other interventions such as microvascular decompression[61].

Table 4 shows patient-centered outcomes across LITT, FUS, and RFA.

## Future directions and synergy

With constant advancements in neurosurgical technology, the future of LITT, FUS, and RFA may extend beyond the current perception of these techniques as competing options. Instead, they may be combined to achieve results superior to what each could accomplish individually. In the following subheadings, four key areas with significant potential for future exploration are outlined.

### Hybrid approaches

Although each of these technologies comes along with its own strengths and limitations, the best outcomes are yielded when they are used together in a complementary fashion. By combining modalities, their benefits may balance each other out, offsetting individual shortcomings. For instance, LITT has been used to treat tumor control in patients who need to resume systemic therapy as soon as reported by Bastos *et al*[67]. On the other hand, FUS has proved its significance in opening the BBB reversibly for extended hours compared to others, making it possible to deliver pharmaceutical drugs that would otherwise not reach the brain[69].

### Precision targeting via AI multimodal imaging

Precision is fundamental in neurosurgery, and emerging advances in AI and Imaging technologies are assisting surgeons in gaining better lesion control. For example, the AI4Neurosurgery system provides real-time guidance during brain tumor surgery, facilitating more precise clinical judgment[70]. Similarly, multimodal imaging combines ultrasound, MRI, and 2D and 3D reconstructions, providing surgeons with a detailed anatomical view during procedures[71]. Together, these tools can increase the safety, speed, and accuracy of minimally invasive techniques like LITT, FUS, and RFA. Moreover, these innovations show potential in solving current challenges, such as treating tumors with irregular margins.

### Personalized neurosurgery

The goal of personalized neurosurgery is to customize the treatment approach to each patient’s specific pathology and circumstances. This method considers both individual values and available resources, in addition to clinical and anatomical factors. For example, MRgFUS is well-suited for patients who want incision-free treatment for essential tremors[72]. In contrast, LITT’s probe-based targeting under MRI guidance is advantageous for patients with small, deep-seated, and hard-to-reach tumors[73]. On the other hand, RFA remains the best option in low-resource settings because of its accessibility and relative ease of use[31].

### From competition to complementary spectrum

**Figure 3.**
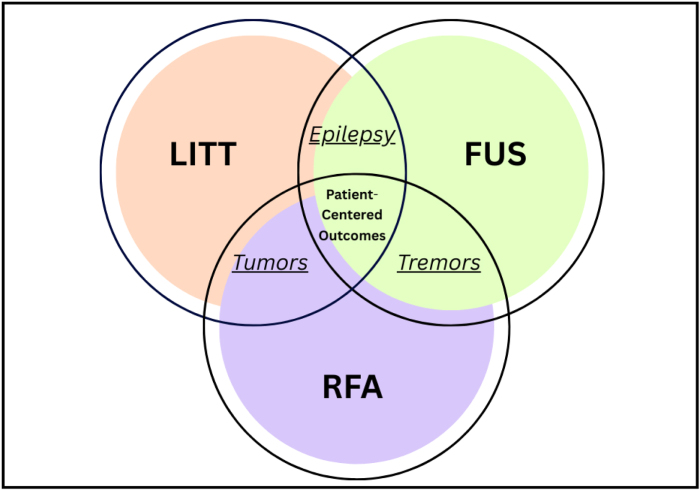
Conceptual model illustrating the shift from competition to complementarity among minimally invasive technologies.

LITT, FUS, and RFA have traditionally been regarded as competing techniques, each aiming to offer the best minimally invasive solution. However, new research suggests that the three modalities may yield better outcomes when viewed as part of a complementary spectrum of care, where each contributes its own unique strengths to the field of minimally invasive neurosurgery. By leveraging the advantages of each modality, such as LITT’s probe-based precision, FUS’s incision-free approach, and RFA’s accessibility, surgeons can tailor treatment strategies to the patient’s individual pathology and circumstances. Figure 3 illustrates this conceptual overlap, highlighting that such a complementary approach has the potential to broaden treatment options and improve patient-centered outcomes in the future of neurosurgery.

## Conclusion

In summary, all three of these approaches are viable alternatives to the traditional methods of neurosurgery. As minimally invasive neurosurgery techniques continue to evolve, the convergence of these three modalities may allow better patient-centered outcomes. Each procedure offers some distinct advantages; for example, LITT, with its laser-focused precision under MRI guidance, has shown promise in treating metastasis and epilepsy. On the other hand, FUS has been applied for the treatment of many movement disorders, including Parkinson’s. Lastly, RFA, with its cost-effectiveness and simpler technology, remains central to epilepsy treatment protocols. As research advances, opportunity exists for these methods to be integrated in complementary ways instead of being competitive with each other. Hybrid strategies could allow a more patient-centered approach, and shortcomings of each may be offset by the strengths of others, thus minimizing damage and broadening therapeutic options. Future direction should focus on tailoring ablation strategies based on tumor characteristics and individual patient profiles. Integration rather than competition will likely expand the neurosurgical toolkit, supporting the development of safer and more effective neurosurgical care.

## Data Availability

All data used in this narrative review are publicly available and sourced from previously published studies. No new data were generated for this work. All included articles have been appropriately cited within the manuscript and are available through the references section.

## Research registration unique identifying number (UIN)

The Research registration unique identification number (UIN) was not required because it is a narrative review.
